# Investigating Manuka Honey Antibacterial Properties When Incorporated into Cryogel, Hydrogel, and Electrospun Tissue Engineering Scaffolds

**DOI:** 10.3390/gels5020021

**Published:** 2019-04-18

**Authors:** Katherine R. Hixon, Savannah J. Bogner, Gabriela Ronning-Arnesen, Blythe E. Janowiak, Scott A. Sell

**Affiliations:** 1 Department of Biomedical Engineering, Parks College of Engineering, Aviation, and Technology, Saint Louis University, St. Louis, MO 63103, USA; katherine.hixon@slu.edu (K.R.H.); savannah.bogner@slu.edu (S.J.B.); gabriela.ronningarnesen@slu.edu (G.R.-A.); 2 Department of Biology, Saint Louis University, St. Louis, MO 63110, USA; blythe.janowiakmulligan@slu.edu

**Keywords:** Manuka honey, tissue engineering, scaffolds, electrospun scaffolds, hydrogels, cryogels

## Abstract

Honey is well-known for its wound healing capability and Manuka honey (MH) contains a unique Manuka factor, providing an additional antibacterial agent. Previously, there has not been a practical way to apply MH to a wound site, which renders treatment for an extended period extremely difficult. Tissue-engineered scaffolds offer an alternative treatment method to standard dressings by providing varying geometries to best treat the specific tissue. MH was incorporated into cryogels, hydrogels, and electrospun scaffolds to assess the effect of scaffold geometry on bacterial clearance and adhesion, as well as cellular adhesion. Electrospun scaffolds exhibited a faster release due to the nanoporous fibrous geometry which led to a larger partial bacterial clearance as compared to the more three-dimensional cryogels (CG) and hydrogels (HG). Similarly, the fast release of MH from the electrospun scaffolds resulted in reduced bacterial adhesion. Overall, the fast MH release of the electrospun scaffolds versus the extended release of the HG and CG scaffolds provides differences in cellular/bacterial adhesion and advantages for both short and long-term applications, respectively. This manuscript provides a comparison of the scaffold pore structures as well as bacterial and cellular properties, providing information regarding the relationship between varying scaffold geometry and MH efficacy.

## 1. Introduction

Honey has been recognized for centuries for its medicinal and healing properties [1,2,3]. Specifically, Manuka honey (MH) has recently become a popular choice due to its unique Manuka factor (UMF) and as a result, its advantageous antimicrobial effects [4,5]. Similar to other honeys, MH has a high sugar content leading to a high osmolality. This sugar alone has been shown to result in antibacterial properties in 61% of honeys [6]. Derived from nectar of the *Leptospermum scoparium* found in New Zealand, MH additionally possesses a low pH ranging from 3.5–4.5. This leads to increased angiogenesis and the stimulation of macrophages [4,7,8,9]. While the hydrogen peroxide released from honey is antibacterial, the key factor in MH that leads to these specific results is the unique Manuka factor (UMF) which provides methylglyoxal (MGO) at the wound site [6,10,11]. Clinically, MH has been shown to successfully treat various bacterium including *Escherichia coli* (*E. coli*), *Enterobacter aerogenes*, *Helicobacter pylori* (*H. pylori*), *Salmonella typhimurium*, and *Staphylococcus aureus* (*S. aureus*) [2].

Despite MH’s blossoming popularity in clinical applications, current use requires a dressing which lacks permanence and results in frequent treatments [12]. In response to this, dressings impregnated with honey have become popular with positive outcomes [13,14,15]. However, novel tissue engineering scaffolds offer a unique avenue for honey delivery while also providing a matrix for guided tissue regeneration [4,16]. Electrospun scaffolds, hydrogels, and cryogels, three very different scaffold types, are appropriate for varying tissue engineering applications due to their difference in structure. Specifically, electrospun scaffolds have commonly been used for skin-related wound healing applications (i.e., burns, pressure ulcers, etc.) due to their flat geometry [17]. However, these scaffolds have exhibited challenges regarding cellular infiltration and mechanical integrity for load-bearing applications [18]. Hydrogels scaffolds have commonly been used for muscle and bone as well as drug and cell-encapsulation; however, these popular scaffolds have inadequate pore sizes for angiogenesis and reduced mechanical stability [17,19]. Cryogel scaffolds have commonly been used for bone and cartilage applications, but are not mechanically strong and exhibit reduced cellular infiltration due to the thickness of the scaffold [19,20]. Previous studies have demonstrated that MH can be incorporated into and released from electrospun scaffolds, hydrogels, and cryogels.

In a study by Minden-Birkenmaier et al [21], electrospun 15 wt% poly(ε-caprolactone) incorporated with 1, 5, 10, and 20% v/v MH resulted in increased osmotic pressure. Additionally, increased fibroblast proliferation and migration along with bacterial clearance of both *Streptococcus agalactiae* and *E. coli* was demonstrated. Another study similarly electrospun various concentrations of MH, but with the polymer silk fibroin [22]. When implanted over a 9 mm wound in BALB/c mice, the wound size was significantly decreased over 12 days due to the presence of the honey. Similarly, silk fibroin has also been incorporated with 5% (v/v) MH to assess the release kinetics and bacterial clearance as affected by differing the UMF [23]. MH has also been electrospun with poly(vinyl alcohol) and likewise exhibited clearance of both *E. coli* and *S. aureus*, as well as an extended release of the anti-inflammatory drug dexamethasone [24,25].

In comparison, hydrogels are hydrophilic, nanoporous gel structures formed through the crosslinking of gel precursors. The resulting three-dimensional (3D) structure can be used for various applications including cell-encapsulation, drug delivery, food additives, wound dressings, and tissue engineering [26,27]. Multiple studies have examined the addition of MH to hydrogel scaffolds for various tissue engineering applications including wound healing and cartilage repair [1,28,29,30]. El-Malek et al [31] incorporated 20% (v/v) MH into a chitosan-gelatin hydrogel, resulting in antibacterial properties to *S. aureus*, *Streptococcus pyogenes*, *Acinetobacter baumannii*, *Pseudomonas aeruginosa*, and *Proteus mirabilis*. Similarly, two studies completed by Bonifacio et al [32,33] examined a MH/gellan gum composite hydrogel which led to the inhibition of both *S. aureus* and *S. epidermidis* clinical isolates. As this was targeting cartilage repair, mesenchymal stem cells were also seeded on the scaffolds and, in addition to no cytotoxic effect, collagen II expression and synthesis of glycosaminoglycans (GAGs) and proteoglycans was observed. The mechanical properties of sodium alginate-based hydrogel films with MH have also been evaluated where the Young’s Modulus was shown to increase with the addition of MH [34].

Similar to hydrogels, cryogels are formed through the crosslinking of a polymer solution. However, following crosslinking, the solution is immediately frozen such that the solvent crystalizes, acting as a porogen. Upon thawing the scaffold, and subsequent melting of the porogen, the resulting structure appears sponge-like and macroporous [19,35]. Such a scaffold can be used for cell separation, chromatography, bioreactors, and tissue engineering techniques [19,36]. Three studies conducted in our lab have incorporated MH into cryogel scaffolds. Silk fibroin has been combined with MH at 1, 5, and 10% (w/v and v/v) and resulted in decreased pore size in comparison to plain silk fibroin cryogels [37,38]. Despite this, MG-63 cells were able to fully infiltrate over 28 days in both studies. Additionally, gelatin cryogels incorporated with MH were also compared where none of scaffolds displayed crack propagation under mechanical loading and led to clearance of both *E. coli* and *S. aureus* after 24 h [38]. Lastly, a study by Hixon et al [23] compared various UMF in 5% (v/v) MH silk fibroin cryogels, concluding that due to the extended release, there was no significant difference in bacterial clearance or adhesion.

Only one study has been found to utilize two separate tissue engineering scaffold techniques to simultaneously assess the release and bacterial clearance of MH [23]. However, this former study focused solely on how the changes in UMF of the honey affected its overall properties. No work has provided a comparison between tissue engineered scaffolds of varying geometry and the affect incorporating honey has on them. This study wishes to compare MH incorporated electrospun scaffolds (ES), hydrogels (HG), and cryogels (CG) to assess how the differences in geometry affect the porosity, bacterial clearance, and cell and bacterial adhesion with respect to MH. These three scaffold types possess a range of geometries and porosities, providing useful tissue-engineered techniques for incorporating MH, which can be tailored according to the desired application.

## 2. Results

### 2.1. Scaffold Characterization

#### 2.1.1. Scanning Electron Microscopy (SEM)

**Figure 1:**
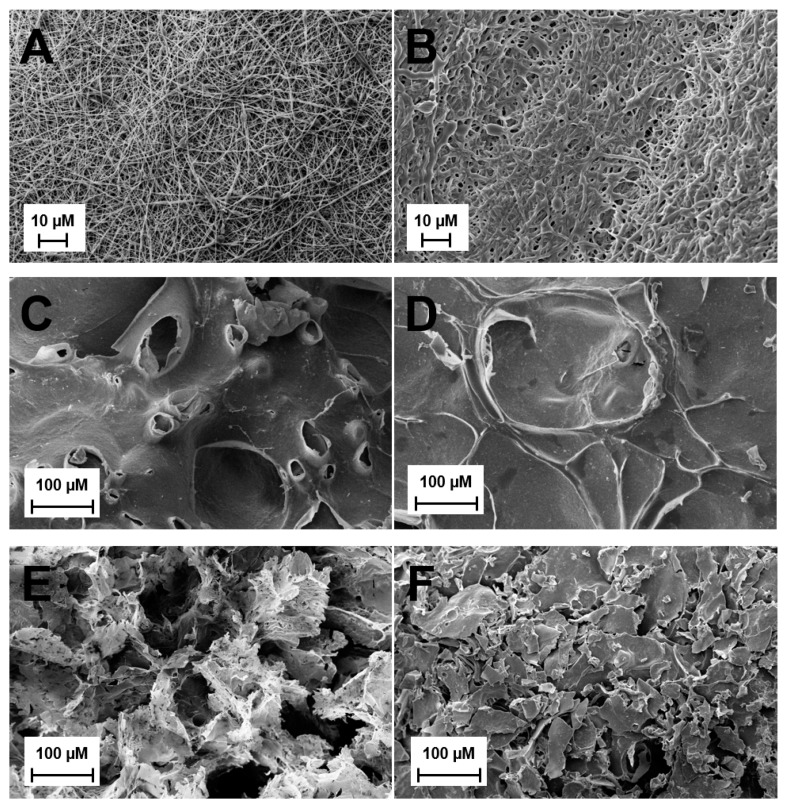
SEM images of (**A**) plain electrospun scaffolds (ES), (**B**) ES + Manuka honey (MH), (**C**) plain hydrogels (HG), (**D**) HG + MH, (**E**) plain cyrogels (CG), and (**F**) CG + MH, obtained under high vacuum. The ES scaffold images were taken at 2000× and both the HG and CG scaffold images were taken at 500×.

SEM imaging was used to observe the pore sizes of ES, HG, and CG scaffolds, ± MH (Figure 1). Visually, the addition of MH resulted in thicker ES fibers that appear to be blended together, with less obvious pores than the plain ES (Figure 1A,B). This is supported in Table 1 where the average fiber diameter of ES + MH scaffolds (0.85 µm) is significantly larger than that of the plain ES scaffolds (0.41 µm; *p* < 0.05). However, with increasing fiber diameter the pore size was shown to decrease resulting in ES + MH scaffolds having a significantly smaller pore area than plain ES scaffolds, also seen in Table 1 (*p* < 0.05). Comparatively, there appears to be no difference in the hydrogel surface, ± MH (Figure 1C,D). Note that preparation for SEM involves lyophilizing the scaffold and, as hydrogels are primarily composed of water, the images are not an accurate representation of the surface of this scaffold [39]. In comparison, the cryogels appear to have a very slight difference in pore size with the addition of MH (Figure 1E,F) where this difference was quantified using µCT in Section 2.1.2.

#### 2.1.2. Microcomputed Tomography

**Figure 2:**
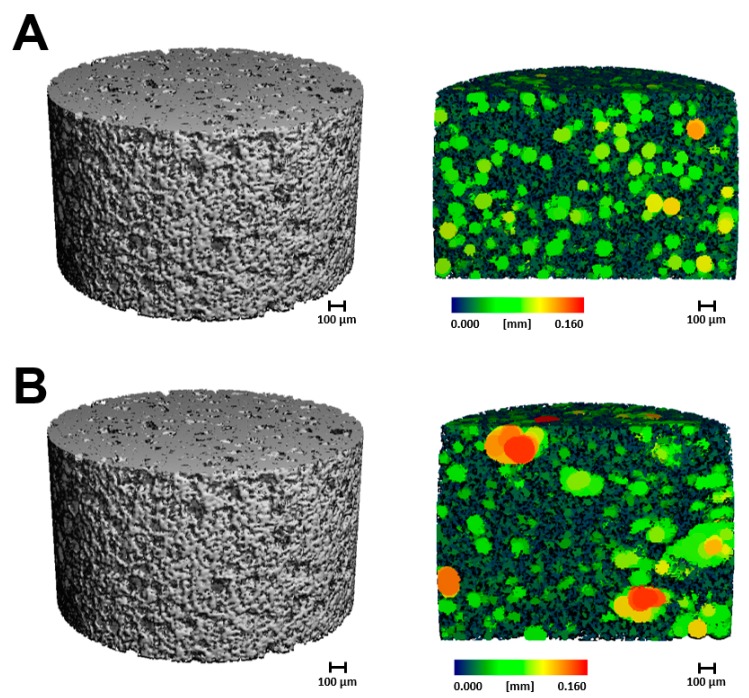
3D reconstruction images of (**A**) plain CG and (**B**) CG + MH. The grey figures display the 3D scaffolds whereas the sagittal cross-section displays the inner pore distribution, with pore size denoted by the color bar.

**Figure 3:**
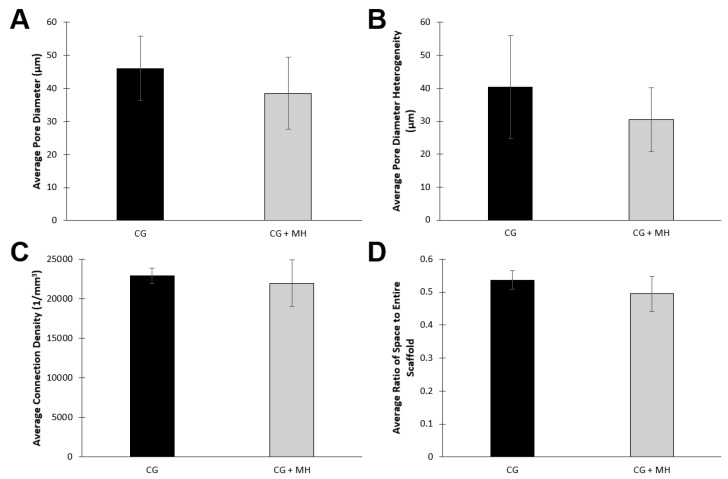
µCT analysis of the CG scaffolds (± MH) providing the average (**A**) pore diameter (µm), (**B**) pore diameter heterogeneity (µm), (**C**) connection density (1/mm^3^), and (**D**) ratio of space to entire scaffold. There was no significant difference between average pore diameter, pore heterogeneity, interconnectivity, and ratio of space to entire scaffold between either of the cryogel types (*p* < 0.05).

All cryogels were also scanned using µCT to provide a quantitative analysis of pore size and interconnectivity (Figure 2 and Figure 3). Note that due to fragility, HG scaffolds were excluded from µCT analysis. Similarly, as the fiber diameters of the ES scaffolds are on the nanoscale and have a very low density, these scaffolds were also excluded from analysis. Figure 2 displays a visual of the scaffold and a cross-section in the sagittal plane. Figure 3 provides a quantitative analysis of the µCT scans of the cryogel. While both the average pore diameter and pore heterogeneity exhibited a slight decrease upon the addition on MH, neither of these values were significant (Figure 3A,B; *p* < 0.05). There was also no significant difference in the average connection density (pore interconnectivity) or ratio of space to entire scaffold between plain CG and CG + MH (Figure 3C,D; *p* < 0.05).

#### 2.1.3. Cellular Adhesion

**Figure 4:**
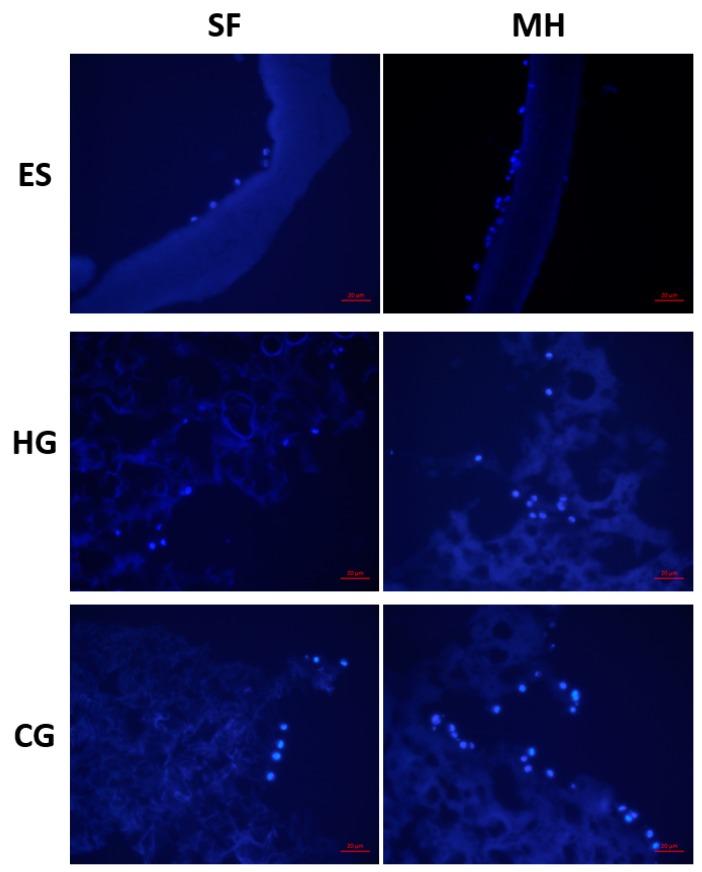
ES, HG, and CG scaffolds seeded with MG-63 cells to assess cellular adhesion. All images were taken at 40× and the scale bar represents 20 μm.

Following cell seeding for two hours, all scaffolds were rinsed and imaged for cell adhesion (Figure 4). For the HG scaffolds, the addition of MH did not have an impact in cell adhesion. In comparison, both the ES and CG scaffolds displayed an increase in cell adhesion to the surface of the scaffolds when MH was present.

### 2.2. Manuka Honey Evaluation

#### 2.2.1. Bacterial Clearance

**Figure 5:**
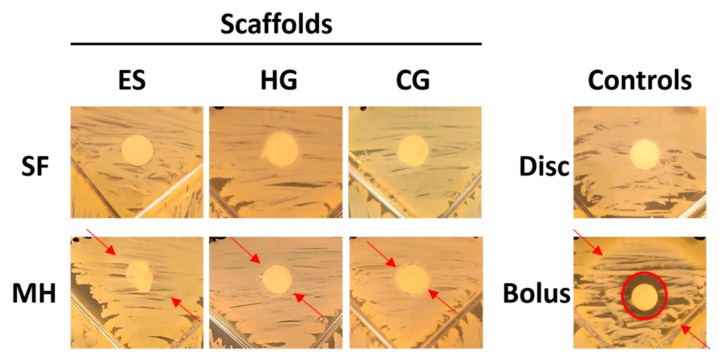
ES, HG, and CG scaffolds (± MH) were placed on plates of *S. aureus* for 24 h to measure both full and partial clearance. The controls include a sterile disc and bolus MH control. Partial clearance attributed to the MH is noted with red arrows while full clearance is circled in red. Note that clearance attributed to the water content of the scaffolds is not marked.

**Figure 6:**
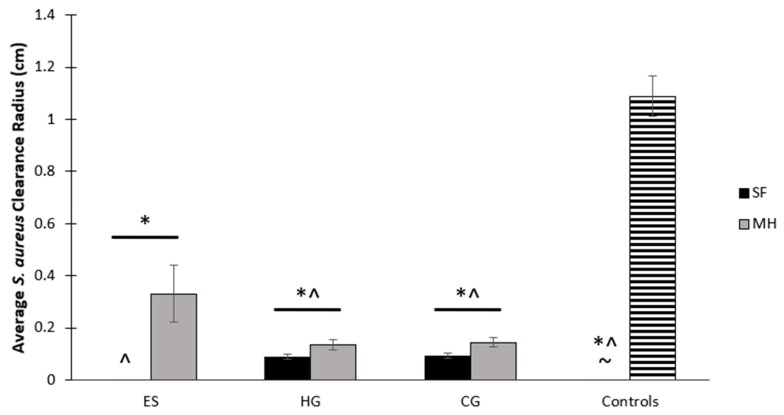
Measurement of *S. aureus* clearance by ES, HG, and CG scaffolds (± MH). Note that for the controls, vertical bars denote clearance by the sterile disc and horizontal bars denote clearance by the bolus of MH. This demonstrates the difference in release of MH based on scaffold geometry, as well as the MH bolus control. The MH bolus control had significantly more full clearance than all scaffolds and the disc control, denoted by *. ES + MH had significantly more partial clearance than all other scaffolds and the disc control, denoted by ^. HG and CG (both ± MH) had significantly more partial clearance than the disc control, denoted by ~ (*p* < 0.05).

All scaffolds were placed on brain heart infusion (BHI) plates spread with *S. aureus* for 24 h (Figure 5). Both full and partial clearance of bacteria was measured from the scaffold to the ring of clearance, providing a measurement of the average clearance radius around the scaffold. The plain HG and CG scaffolds all had a small amount of clearance due to their water content; however, those containing MH had a wider partial clearance that spanned outside of the moisture ring (Figure 5 and Figure 6). The ES + MH scaffolds resulted in the most partial clearance (0.33 cm) and, as ES scaffolds are not filled with water, none of this clearance was attributed to moisture release (Figure 6). Specifically, ES + MH scaffolds had significantly more partial clearance than all other scaffolds and the disc control (*p* < 0.05). The sterile disc control exhibited no clearance, but the bolus of MH, with the largest full clearance of 1.09 cm, had significantly more clearance than all scaffolds and the disc control. Finally, HG, HG + MH, CG, and CG + MH all had significantly more partial clearance than the disc control (*p* < 0.05).

#### 2.2.2. Bacterial Adhesion

**Figure 7:**
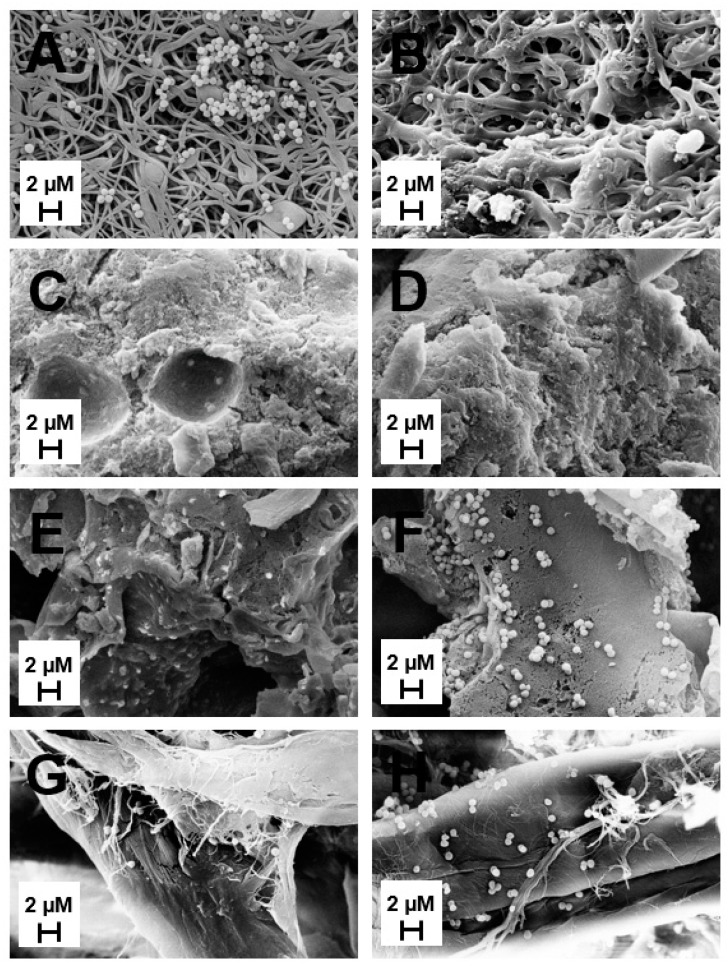
SEM images of bacterial adhesion for (**A**) plain ES, (**B**) ES + MH, (**C**) plain HG, (**D**) HG + MH, (**E**) plain CG, (**F**) CG + MH, (**G**) sterile disc control, and (**H**) vancomycin control. All images were taken at 15 kV with a scale bar of 2 µm.

**Figure 8:**
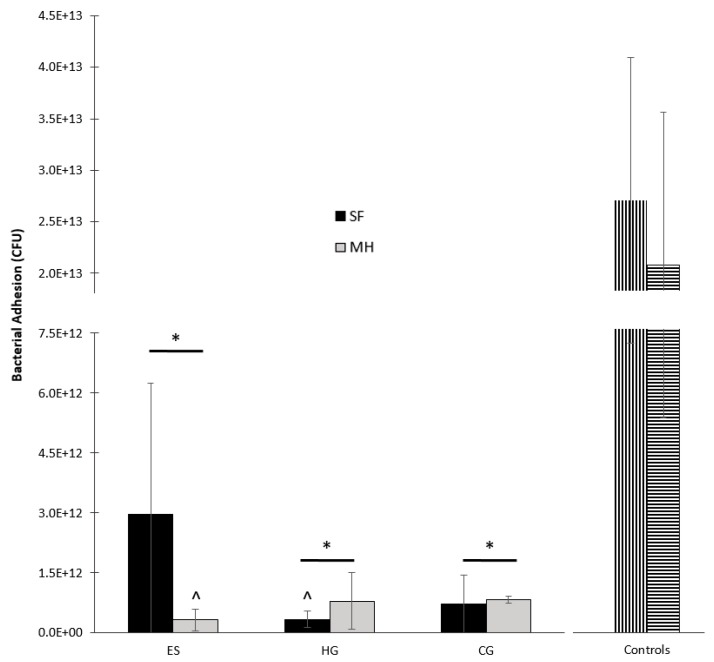
The adhesion of *S. aureus* to ES, HG, and CG scaffolds (± MH) and controls. Note that for the controls, vertical bars denote adhesion to the sterile disc and horizontal bars denote adhesion to the vancomycin disc. The sterile disc control had significantly more adhesion than all scaffolds (± MH), denoted by *. The vancomycin disc control had significantly more adhesion than ES + MH and HG scaffolds only, denoted by ^ (*p* < 0.05).

The adhesion of *S. aureus* to all scaffold types was both imaged and quantified following four hours of submersion in bacterial culture (Figure 7 and Figure 8). The plain ES scaffold displayed a large amount of bacterial adhesion whereas the addition of MH resulted in reduced adhesion (Figure 7A,B and Figure 8). However, both HG and CG cryogels displayed similar *S. aureus* adhesion, regardless of the presence of MH (Figure 7C–F and Figure 8). Finally, the vancomycin disc control had slightly less adhesion than the sterile disc control, where the sterile disc had significantly more *S. aureus* present than all scaffold types (Figure 7G,H, *p* < 0.05). Comparatively, the vancomycin disc control only had significantly more *S. aureus* adhesion than ES + MH and HG. There was no significant difference in *S. aureus* adhesion between any of the scaffold types, regardless of the presence of MH (*p* < 0.05).

## 3. Discussion

This study evaluated three varying types of tissue-engineered scaffolds (ES, HG, and CG) with and without the addition of MH. Previous work has demonstrated the ability to dope these scaffolds with MH, but no work has compared the varying effects on scaffold porosity, bacterial clearance, and bacterial and cellular adhesion. Due to the difference in fabrication techniques, these silk fibroin (SF)-based scaffolds are physically different, ranging from nanoporous fibrous mats (ES scaffolds) to macroporous sponges (CG scaffolds). Thus, it is of interest to assess and provide a side-by-side comparison of the effects of MH on the scaffold’s pores and physical structure, as well as the variations in release on bacteria.

Initial analysis used SEM and µCT to provide both a qualitative and quantitative assessment of the different scaffold types and any variations with the addition of MH. Figure 1A demonstrates the fibrous mat of the ES scaffolds compared to the macroporous structure of the CG (Figure 1E). With the addition of MH, the ES scaffolds experienced a clumping of fibers and overall increase in fiber diameter which, consequently, decreased pore area, as previously demonstrated (Figure 1A,B) [1,40]. While it appears that the CG pores decreased with the addition of MH (Figure 1E,F), analysis by µCT did not find a significant difference in the average pore diameter, heterogeneity, or connection density (Figure 3; *p* < 0.05). While previous work had found a significant decrease in pore diameter, these measurements were acquired by ImageJ which relies on a dehydrated section of the scaffold for analysis [37]. Conversely, µCT provides a 3D measurement of the hydrated structure (Figure 2 and Figure 3) [23,41]. As noted previously, HG scaffolds are primarily composed of water and thus, SEM images do not provide an accurate representation of the scaffolds surface; however, the incorporation of MH did not visually alter the structure (Figure 1C,D). Future work could utilize liquid nitrogen for the dehydration process, as well as environmental SEM or optical microscopy following cryosectioning to improve visualization. Additionally, a more homogeneous analysis method should be identified to provide porosity quantification that can be compared across all tissue engineered scaffold types.

Due to the described changes in porosity and surface topography, it is of interest to evaluate the potential for cellular attachment. This study found that all tissue-engineered scaffold types supported cellular adhesion after only two hours of incubation (Figure 4). While the HG scaffolds did not display a difference in adhesion with the addition of MH, both ES and CG scaffolds had an increase in adhesion when MH was incorporated. SF is known to contain cell attachment sites which, in this study, supported adhesion on all scaffolds [23]. Previous work has demonstrated that, in vitro, MH concentrations as low as 5% is cytotoxic for cells, attributed to the acidic pH in a closed-off environment [7]; however, due to the slow release and thus, lower quantity of MH released from the scaffolds, this does not occur with the ES, HG, or CG scaffolds. This study replicated the scaffold fabrication protocols from a previous body of work in which glucose release was evaluated over 14 days [23]. Here, it was shown that 0.5 mg/mL of glucose was released from both ES and CG scaffolds for the first 24 h which subsequently decreased over the tested time period. This demonstrates the sustained MH release, as compared to a bolus of honey incorporated directly into the media. In a study by Hixon et al [38], MH was shown to increase cellular infiltration, supporting the increased adhesion demonstrated in this study. Note that previous work has demonstrated that, despite the differences in pore size with the addition of MH, cells are able to infiltrate and proliferate through all scaffold types, suggesting that following successful adhesion, cells would migrate throughout the scaffold and support tissue formation [22,37,38,42]. As there was a slight difference in SF concentration between the scaffold types, it is possible that this could influence scaffold structure and cell adhesion moieties; however, based on the data, the authors do not believe this difference to be significant as similar cell adhesion occurred prior to the addition of MH. Future work should focus in more detail on quantifying cell compatibility, adhesion, and infiltration potential, for example, through the examination of cytoskeletal proteins to further assess cell morphology. Additionally, a wide variety of cell types could be analyzed to provide an assessment of these different scaffold types compatibility for various tissue engineering applications.

Finally, the major benefit of MH in tissue engineering applications is its potential for bacterial clearance and inhibition. Previous work has demonstrated the clearance of various bacterium including *S. aureus* [2]. This study chose to examine both the potential for clearance and adhesion, as well as the differences between scaffold types based on geometry. Following 24 h of incubation on *S. aureus*, the ES + MH scaffolds exhibited the highest partial clearance (Figure 5 and Figure 6). This is due to the ES geometry being composed of a flat mat of fibers which concentrates the MH at the surface of the scaffold and leads to an immediate amplified release. In comparison, both the HG and CG scaffolds possess a 3D geometry where the MH is distributed throughout, resulting in an extended release which has previously been shown to last between two and four weeks [38,43]. Previous work has suggested that increasing surface roughness results in greater cell adhesion while others demonstrate the opposite, where there is decreased adherence. Additionally, differences in patterned surfaces has also been shown to decrease bacterial adhesion [44]. In combination with the variations in geometry and presence of Manuka honey, it was anticipated that the tissue-engineered scaffolds in this study would exhibit differences in bacterial adhesion. While the ES scaffolds were the only scaffold to have a significant decrease in the degree of bacterial adhesion with the addition of MH, this may also be due to the difference in geometry (Figure 7 and Figure 8). With a fast release of MH, *S. aureus* was unable to adhere to the surface as readily as plain ES scaffolds. As both the HG and CG scaffolds have an extended MH release, less honey is released at each time point and the *S. aureus* can adhere more readily. Both release rates provide benefits for various tissues and applications based on the structural and antibacterial need. Future work could focus on the ratio of live and dead bacteria following adhesion to the scaffolds to further quantify the effects of MH. 

Overall, this study provided an overview of the capability of incorporating MH in ES, HG, and CG scaffolds. It also demonstrated the effects of the additive on the scaffolds themselves, as well as the differences in *S. aureus* clearance and adhesion based on geometry and its overall effect on cells.

## 4. Materials and Methods

### 4.1. Scaffold Fabrication

All scaffolds were fabricated using silk fibroin (SF) from Bombyx mori cocoons, where the liquid SF was obtained as described previously [45].

#### 4.1.1. Electrospun Scaffolds

Five percent SF electrospun (ES) scaffolds were fabricated, with and without 5% MH. For the ES scaffolds that included honey, 5% MH (Manuka Honey, Medical Grade 12+, Ndal Laboratories, Monterey, CA, USA) was dissolved in 1,1,1,3,3,3-hexafluoro-2-propanol (HFP, Oakwood Chemical, West Columbia, SC, USA) using a 20 min water bath sonication (Branson 200 Ultrasonic Cleaner, Branson Ultrasonics, St. Louis MO, USA). Following dissolution, 5% lyophilized SF was placed in the solution and allowed to dissolve overnight under mechanical agitation. The following day, the solution was loaded in a 5 mL syringe fixed with a blunt-tipped 18 gauge needle (PrecisionGlide, Becton Dickinson, Franklin Lakes, NJ, USA). The solution was then electrospun under the following parameters: voltage of 23 kV, working distance of 16.5 cm, and extrusion rate of 3 mL/h. The fibers were collected on a translating, rotating (400 rpm) rectangular (0.5 cm × 2.5 cm × 9 cm) stainless steel mandrel. Following removal, all ES scaffolds were crosslinked using methanol vapor and stored at room temperature (RT) in a desiccation chamber. Note that for the ES scaffolds that did not include honey, the initial sonication step was excluded from the solution preparation.

#### 4.1.2. Hydrogels

The 4.5% SF hydrogels (HG) were fabricated per the protocol developed in our laboratory, described previously [37]. Five percent MH was dissolved in the prepared aqueous SF solution by a mechanical spinner. Once dissolved, 0.5 mL of the solution was placed in a 2 mL rounded-bottom microcentrifuge tube. A sonication probe (probe intensity of 2, Fisher Sonic Dismembrator Model 100, Pittsburgh, PA, USA) was placed in the aliquot and sonicated for 30 s. The tube was then gently tapped to remove air bubbles and left at RT for gelation. Plain SF HG that did not include MH were immediately sonicated without the dissolution step.

#### 4.1.3. Cryogels

SF cryogels (CG), with and without MH, were prepared similarly to the above HG fabrication protocol [37]. However, following the dissolution of MH, the solution was cooled at 4 °C for one hour prior to sonication. During the sonication process, a precooled microcentrifuge tube was placed in a small beaker of ice water to prevent any heating. Following sonication and the removal of any bubbles, the tube was immediately placed in a stirred methanol bath at −20 °C for gelation. After 24 h, the CG were removed and thawed in RT deionized (DI) water for an additional 24 h. Note that plain SF CG were created by skipping the MH dissolution step.

### 4.2. Scaffold Characterization

#### 4.2.1. Scanning Electron Microscopy

Scanning electron microscopy (SEM; Zeiss, Evo LS15, Oberkochen, Germany) was used to visualize the pores of all scaffold types. Following preparation, all HG and CG scaffolds were frozen at −80 °C for one hour and then lyophilized for 24 h. All scaffolds (*n* = 3) were then cut to display a cross-section of the pores and mounted on an aluminum stub. The stubs were sputter coated (SoftComp, Bal-Tec SCD 005, München, Germany) in gold at 20 mA for 360 s. SEM images of the HG and CG scaffolds were taken at 500× and the ES scaffolds were taken at 2000× at an operating voltage of 5 kV. The pores of the ES scaffolds were analyzed using ImageJ (NIH), as previously described [41]. 

#### 4.2.2. Microcomputed Tomography

Microcomputed tomography (µCT) was used to quantify the pore size and interconnectivity of the CG scaffolds (*n* = 3). µCT (µCT 35, Scanco Medical, Wayne, PA, USA) scans were completed on hydrated samples using the following parameters: X-ray tube potential 45 kVp, X-ray intensity 4 W, isotropic voxel size 7 µm, integration time 600 ms, frame averaging 1, projections 500, and medium resolution scan. Pore analysis and interconnectivity data was obtained using the manufacturer installed trabecular morphology analysis. Voxels above a threshold of 80 per milles (determined through pilot testing) were considered scaffold and those below 80 per milles were considered empty space, as previously described [41].

#### 4.2.3. Cellular Adhesion

Cellular adhesion was used to demonstrate the cell compatibility with the various scaffold types. The ES, HG, and CG scaffolds (*n* = 3) were sterilized in peracetic acid for 90 min and then rinsed with sterile phosphate buffered solution (PBS) for 10 min, three times. Following sterilization, all scaffolds were placed in a 48 well plate (Falcon, Fisher Scientific UK Ltd, Loughborough, UK), surrounded by cloning rings (Fisher Scientific). 100 µL of 50,000 human bone osteosarcoma-derived cells (MG-63, passage 4; ATCC) suspended in DMEM with 4.5 g/L Glucosenand l-Glutamine, 10% FBS, and 1% penicillin-streptomycin was seeded onto each scaffold through the dropwise method, as described previously [38,46]. All scaffolds were then incubated for one hour at 37 °C and 5%, at which time an additional 300 µL of complete media was added to each well. The plates were placed in the incubator for an additional hour to allow for attachment. At this time, all scaffolds were moved to a new well-plate and rinsed with sterile 1× PBS twice, 5 min each, under mechanical agitation to ensure that any cells that had not adhered to the surface of the scaffolds were removed. All scaffolds were removed and immediately fixed in formalin (Fisher Scientific) for storage for at least 24 h. In preparation for sectioning, all scaffolds were soaked in 30% sucrose (Acros, Molinons, France) for 24 h, embedded in Tissue Freezing Medium (Triangle Biomedical Sciences, Fisher Scientific), and frozen at −80 °C overnight. The scaffolds were then cryosectioned at 20 mm and stained with 40,6-diamidino-2-phenylindole, dihydrochloride (DAPI, 98%; Acros). Images were taken by an optical light microscope (Zeiss, Axiovert 200) at 40×. Note that MG-63 cells were chosen due to their popularity in both hydrogel and cryogel tissue engineering applications to demonstrate basic adhesion and other cell types should be examined in future studies [19,47,48,49,50]. This early timepoint for adhesion was chosen as previous work has already examined later timepoints including 1, 4, 7, and 28 days [21,38]. As MH release has been shown to be highest in quantity during the first 24 h, this early timepoint was most appropriate for identifying the immediate effect on cellular interaction [23].

### 4.3. Manuka Honey Evaluation

#### 4.3.1. Bacterial Clearance

A freshly prepared isolated colony from a BHI plate of *Staphylococcus aureus* subsp. *aureus* Rosenbach (*S. aureus*; ATCC: 12600) was chosen to represent a Gram-positive bacterial strain. The bacteria was spread on BHI plates using a sterile swab and 6 mm punches of the ES, HG, and CG scaffolds, ± MH, were placed in the center of each of the quadrants (*n* = 3). Additionally, sterile discs were used as controls, with and without the addition of a bolus of MH. The plates were then incubated at 37 °C for 24 h. Images of the clearance were taken and measured using ImageJ (NIH). To find this, the average diameter of the scaffold/sterile disc was subtracted from the average diameter of the clearance. This value was then divided by two to provide the distance cleared from the scaffold/disc (cm), as previously described [23]. Note that both partial and full clearance were quantified where partial clearance had less bacteria in comparison to the lawn of bacteria while full clearance occurred when no bacteria remained.

#### 4.3.2. Bacterial Adhesion

All scaffolds were also incubated in *S. aureus* to demonstrate short-term bacterial adhesion, as described previously [23,38]. Briefly, an overnight culture of *S. aureus* was created using 50 mL of BHI. All scaffold types were placed in untreated 24 well plate (Fisher Scientific) with 2 mL of the bacterial culture. These plates then underwent mechanical agitation for four hours at 37 °C, at which time all scaffolds were removed. Each scaffold was then rinsed twice with 1× PBS to remove any non-adherent bacteria. Some scaffolds were immediately placed in formalin for SEM imaging. The remaining scaffolds were placed in a microcentrifuge tube filled with 1 mL of 1× PBS. All tubes were continuously vortexed for 30 min to remove all adhered bacteria. Following this, 100 µL aliquots were serially diluted on BHI ager plates to quantify the adhered bacteria (CFU/mL). Note that the scaffolds for SEM were prepared through dilution in graded alcohol 30, 50, 70, 80, and 90% (15 min) and 100% (1 h), followed by critical point drying (CPD 030 Critical PointDryer, Niesgrau, Germany). Sterile discs and discs containing 5 mg of vancomycin (Becton Dickinson, Franklin Lakes, NJ, USA) served as controls for this study.

### 4.4. Statistical Analysis

Statistical analysis was determined using SPSS software (IBM) with a statistical significance determined at an alpha value of 0.05 for all tests. Differences were determined through appropriate Tukey-post-hoc analysis, evaluating significance.

## 5. Conclusions

Honey has been a target for bacterial inhibition and wound healing for centuries, experiencing a resurgence in popularity following the development of antibiotic resistance strains. This study chose to study three scaffolds formed through varying fabrication techniques (ES, HG, and CG scaffolds) and their impregnation with MH. While the incorporation of MH into varying scaffold fabrication techniques has previously been explored individually, no study to date has removed material differences and focused primarily on scaffold geometry and its impact on the overall effects of MH integration and release from the scaffolds. ES scaffolds were noted for their rapid MH release which resulted in significantly more partial bacterial clearance than other scaffold geometries (i.e., CG and HG formulations). These scaffolds also demonstrated a reduction in adhered bacteria, compared to control disks and other MH-containing scaffold geometries. This would indicate that despite the high surface area of the ES scaffolds which would lend itself to rapid bacterial colonization, the integrated MH was readily exposed on the surface of the generated fibers and reduced adhesion. This was a different response from the HG and CG scaffolds, which had a less immediate and noticeable impact on clearance and adhesion. We anticipate that the more-sustained release of MH here was due to its integration within the polymer networks of the scaffolds rather than being bound to a fiber surface. Future work should further explore cellular compatibility with a variety of cell types, as well as extended timepoints following adhesion to note the potential for cellular infiltration and colonization for the various scaffolds. All tissue-engineered scaffold types offer benefits for different wound healing applications, and this manuscript provides an overview of scaffold properties as well as the potential for bacterial clearance and cell adhesion as varied by scaffold geometry.

## Figures and Tables

**Table 1:** Average fiber diameter, pore diameter, and pore area of electrospun scaffolds (ES) scaffolds ± Manuka honey (MH) obtained from SEM images using ImageJ. ES + MH scaffolds had a significantly larger average fiber diameter (µm) and a significantly smaller average pore area (µm^2^) than the plain ES scaffolds, denoted by * (*p* < 0.05).

Scaffold	Average Fiber Diameter (µm)	Average Pore Diameter (µm)	Average Pore Area (µm^2^)
ES	0.41 ± 0.21 *	3.28 ± 1.55	23.98 ± 17.76 *
ES + MH	0.85 ± 0.40 *	3.80 ± 1.85	16.00 ± 10.70 *
